# Rethinking public health in the digital food era

**DOI:** 10.1186/s12966-026-01920-1

**Published:** 2026-05-28

**Authors:** Adyya Gupta, Kylie Fraser, Adrian J Cameron

**Affiliations:** 1 Deakin University, Geelong, Australia, Deakin Centre for Global Preventive Health and Nutrition, Deakin Institute for Health Transformation, School of Health and Social Development, Faculty of Health, Geelong, Australia; 2 Deakin University, Geelong, Australia, Deakin’s School of Exercise and Nutrition Sciences, Faculty of Health, Geelong, VIC Australia

## The online food retail environment – a public health challenge and opportunity

Advancements in digital technologies have transformed how food is accessed, with food choices increasingly made online rather than in physical stores. Online food retail environments including online grocery services (OGS), online food delivery services (OFDS), and meal kit subscription services (MKSS), have experienced enormous growth since the COVID-19 pandemic [1]. Widespread use of smartphones and internet access, combined with the convenience of food delivery and the changing patterns of work and eating out, are among the key drivers responsible for the rapid expansion of these food environments [2]. In 2025, approximately 2.5 billion people (mostly 15–35 years) worldwide ordered food using these online food retail services [1]. New evidence indicates that unhealthy foods (those high in sugar, salt and fat) are preferentially promoted on OFDS and OGS and to some extent on MKSS [3].

At the recent 2025 International Society of Behavioural Nutrition and Physical Activity (ISBNPA) Conference in Auckland, New Zealand, our work highlighted the urgent need to better understand the role of online food retail environments in shaping food choices and health outcomes. The global proliferation of online food platforms, driven by increasing demand, means that understanding their potential impact on diets and health at a population level is critical. While making life more convenient, which may be particularly important for very busy people, households with children, or those with mobility restrictions, online food retailers are also making unhealthy foods more accessible, and desirable through extensive promotions [3]. There is currently very little evidence for the implications of the shift to acquiring food from online food retail on diets, health outcomes and equity, or how online food retail platforms can be harnessed for socially desirable outcomes. This commentary summarises key challenges and opportunities, particularly the need for a collaborative approach to creating health-enabling online food retail environments.

## Challenges for public health policy

Online food retail environments represent a challenging setting for public policy. Examining online food environments through a Commercial Determinants of Health lens reveals how profit-driven marketing practices systematically drive unhealthy food purchases and amplify health inequities [4]. Digital marketing techniques are highly personalised, using data on customer demographics, geographic location and online browsing and purchase history. Targeted to the individual and powered by multinational food companies and fast-food chains, they are increasingly used to nudge customers towards unhealthy food choices. These tactics include aggressive price promotions and cross-platform advertising across websites, mobile apps and social media [3]. The algorithms behind OFD platforms repeatedly recommend consumers previously purchased food items and tag specific unhealthy food items as popular to enhance their visibility and appeal. While evidence shows that this disproportionate promotion of unhealthy food increases purchasing in the short term, it is also likely that this reinforces lifelong unhealthy eating patterns [5]. This is particularly of concern among young people who may be at a high risk of exploitation due to their social and economic circumstances. Through targeted and personalised marketing messages, OFDS and OGS persuade consumers to purchase and consume unhealthy food products [3]. With newly found purchasing power and autonomy to make their own food choice decisions, but with limited money of their own, young people are more likely to select food on price promotion, which our research has shown is typically unhealthy [2, 6]. Furthermore, the lack of clear and easily accessible nutrition information online also places a greater burden on customers with limited food literacy, thus deepening health inequalities.

Another barrier to policy and practice change is the profit-driven strategies of the online food industry. This includes lobbying of decision makers, strategic partnerships, and shifting the framing of public discourse to individual responsibility for food choices, deflecting attention from corporate accountability. A recent case study of Uber Eats Australia [4] revealed a strategic corporate approach aimed at shaping public perception and consolidating market power. The company framed itself as being of economic importance to Australia, creating a positive public image that can help to distract public from the health risks related to the foods sold on their platforms. Similar reputational management practices have been observed by this sector in other countries [7, 8]. Compared to OFDS and to some extent the OGS, commercial MKSS are marketed as healthy alternatives to ready-to-eat food which may be part of the reason for their increasing popularity and global expansion. Such marketing of MKSS may have resulted in a perception that meal kits are inherently healthy. This has led to new research evaluating the impact of non-commercial MKSS on health and dietary outcomes [9, 10].

While examples of regulating the promotion and placement of unhealthy foods in physical stores are beginning to appear in the United Kingdom [11], the lack of access to algorithmic data that drives personalised marketing on online food retail platforms makes it extremely difficult to develop policies and monitor impacts. The challenge of collecting personalised data on exposure to marketing of unhealthy food options on online food retail platforms also limits the ability to determine whether the use of online food retail results in customers making more unhealthy choices than they already do in physical food stores. Adding to these challenges, there is considerable ambiguity regarding who is responsible or accountable for providing clear nutritional guidance on OFDS – is it the third-party online platform or the food outlet registered on that platform? Online food retail platforms thus operate in a regulatory grey zone, with the technology and platforms evolving faster than policy frameworks can keep up with.

### Opportunities for public health innovation

In recognition of these challenges, the WHO developed a monitoring framework (2019) [12] to address digital marketing of unhealthy food and beverages to children. It provides guidance for governments and key stakeholders on ways to monitor, regulate, and reduce children’s exposure to harmful marketing online. Despite this, the public health policy responses remain slow, fragmented, and reactive as the evidence of a clear causal link between the food purchased using online food retail platforms and its health impacts is yet to be established.

While online food retail platforms pose challenges, they are dynamic, scalable and deeply embedded in everyday life, making them potentially powerful avenues for promoting healthier food choices and public health innovation. The online food sector can adopt a range of health promoting practices to encourage restaurants to increase the healthfulness of their food offerings [13]. These include restructuring of digital interfaces to nudge consumers towards healthier food purchases, altering the assortment of food products to display healthier foods as the default, re-focusing marketing techniques to display promotions on healthy food items on the menu and providing recommendations on healthy food swaps that meet the country specific dietary guidelines. OFDS could also partner with healthier restaurants and incentivise restaurants that offer healthier products (e.g. use of healthier cooking oils). This can further encourage restaurants to adopt innovative ways to create healthier menus. For OGS, supermarkets could provide interpretive health information for all products available, even where this information is not provided on packages by manufacturers. An example is the voluntary Australian and New Zealand Health Star Rating System which is missing from many packaged food products sold directly on OGS [14]. With regards to MKSS, the service providers could better align the nutritional composition of the meals with dietary guidelines by modifying recipes to reduce added sugar, salt and fat and increase dietary fibre [15]. These proposed modifications across online food retail platforms have the potential to contribute towards the prevention of diet-related chronic disease.

## A call to action- directions for public health scholarship and practice

Action against the powerful influence of commercial actors requires collective, cross-sectoral commitment to reimagine online food environments for public good. Governments must step into active stewardship roles—setting clear definitions, nutrition standards, regulating harmful digital marketing, and fostering innovation that prioritises public health over profit. With support from the government, public health experts could generate evidence on new ways to monitor marketing of food and beverages sold in online food environments. This evidence will be imperative for governments to hold companies accountable on their marketing practices. Furthermore, this evidence should be generated for all three online food retail platforms as misaligned actions can undermine public health and create compliance gaps. Beyond documenting harms (which is crucial in its own right for advocacy and accountability), researchers and behavioural nutrition practitioners could work with service providers to co‑design and test the effectiveness and the commercial feasibility of health promoting changes on online food retail platforms. Through this type of partnership, researchers and practitioners can develop (a) new benchmarking frameworks that include the role of service partners in co-creating and sustaining health promotion interventions and (b) implementation guides for product managers and menu partners for easy modifications and to create healthy defaults. As emphasised at ISBNPA 2025, taking care of people and creating an environment that sustains them into the future requires courageous leadership, shared responsibility, and a commitment to reimagining the online food environment as a space for health, equity, and sustainability. We envision a future where online food environments support equitable access to nutritious foods, empower informed choices, and foster health-enabling food systems. The challenge is urgent—but so is the opportunity.

## Data Availability

No datasets were generated or analysed during the current study.
